# Novel Technique of Transepithelial Corneal Cross-Linking Using Iontophoresis in Progressive Keratoconus

**DOI:** 10.1155/2016/7472542

**Published:** 2016-08-15

**Authors:** Marco Lombardo, Sebastiano Serrao, Paolo Raffa, Marianna Rosati, Giuseppe Lombardo

**Affiliations:** ^1^Fondazione G.B. Bietti, IRCCS, Via Livenza 3, 00198 Roma, Italy; ^2^Dipartimento di Medicina Molecolare, Università di Padova, Via A. Gabelli 63, 35121 Padova, Italy; ^3^Consiglio Nazionale delle Ricerche, Istituto per i Processi Chimico-Fisici, Viale Stagno d'Alcontres 37, 98158 Messina, Italy; ^4^Vision Engineering Italy srl, Via Adda 7, 00198 Rome, Italy

## Abstract

In this work, the authors presented the techniques and the preliminary results at 6 months of a randomized controlled trial (NCT02117999) comparing a novel transepithelial corneal cross-linking protocol using iontophoresis with the Dresden protocol for the treatment of progressive keratoconus. At 6 months, there was a significant average improvement with an average flattening of the maximum simulated keratometry reading of 0.72 ± 1.20 D (*P* = 0.01); in addition, corrected distance visual acuity improved significantly (*P* = 0.08) and spherical equivalent refraction was significantly less myopic (*P* = 0.02) 6 months after transepithelial corneal cross-linking with iontophoresis. The novel protocol using iontophoresis showed comparable results with standard corneal cross-linking to halt progression of keratoconus during 6-month follow-up. Investigation of the long-term RCT outcomes are ongoing to verify the efficacy of this transepithelial corneal cross-linking protocol and to determine if it may be comparable with standard corneal cross-linking in the management of progressive keratoconus.

## 1. Introduction

Corneal cross-linking is an established procedure aimed at slowing down or halting keratoconus progression [1]. The efficacy of the conventional riboflavin/UV-A irradiation procedure was primarily demonstrated by laboratory studies suggesting that it increases the biomechanical strength of the treated cornea. In addition, several clinical studies have evidenced how the treatment is effective in slowing down or halting the progression of keratoconus up to 10 years of follow-up [2–9].

The conventional corneal cross-linking procedure includes the removal of corneal epithelium to permit adequate penetration of riboflavin in the stroma. Epithelial removal, however, is responsible for most of the major cross-linking related complications, which include postoperative pain, vision impairment, and risk of infection. Major efforts have been dedicated to overcoming the epithelial barrier to riboflavin penetration and reducing treatment time, with the aim of maintaining efficacy and improving safety of the treatment [10]. On the other hand, there are still controversies on how riboflavin may penetrate in the stroma through an intact epithelium or how the epithelium may limit UV-A irradiation of the stroma soaked by riboflavin.

It has been widely shown that dextran-enriched solutions greatly limit the penetration of riboflavin in the stroma through the intact epithelium [10, 11]. Recently, Shalchi et al. [12] have revised a series of peer-reviewed papers comparing the results of standard cross-linking (total of 45 papers) and transepithelial cross-linking (total of 5 papers) in the management of progressive keratoconus. Although transepithelial corneal cross-linking has been shown to be safe without any related epithelial wound healing complication, 75% of the cases have shown a continued keratoconus progression one year after treatment, whereas the majority of studies on standard cross-linking (≥90%) have shown reduction in maximum simulated keratometry in the same period. The only study that has shown comparable results between transepithelial and standard corneal cross-linking did not use dextran-enriched riboflavin solution for the transepithelial treatment [12].

Overall, the limited number of published outcomes from randomized controlled trials (RCTs) makes it challenging to draw clear conclusions on the efficacy of the many techniques used for transepithelial corneal cross-linking [13–20]. Robustly designed controlled trials are required to provide accurate results between techniques. Currently, five RCTs (NCT02117999, NCT02456961, NCT02349165, NCT01181219, and NCT01868620) are aiming to compare the results of transepithelial corneal cross-linking with the standard procedure for stabilization of progressive keratoconus; three of these trials (NCT02117999, NCT02456961, and NCT01868620) are using dextran-free riboflavin solutions to moisten the corneal stroma through the intact epithelium using iontophoresis.

Iontophoresis is a noninvasive technique used to deliver a charged substance transcorneally by repulsive electromotive force using a small electrical charge applied to an iontophoretic chamber. In* ex vivo* studies, we have assessed the diffusion of riboflavin in dextran-free 0.1% hypotonic solution enriched with ethylenediaminetetraacetic acid (EDTA) and trometamol in the corneal stroma of eye bank donor eyes and the effect of rapid UV-A irradiation of the cornea (i.e., 10 mW/cm^2^ for 9 minutes) after transepithelial soaking using iontophoresis. Experimental work has demonstrated that though the stromal concentration of riboflavin after iontophoresis was lower than conventional soaking, the stiffening effect on the anterior cornea was almost comparable to that of standard cross-linking [21, 22]. Other laboratory studies have shown that iontophoresis is effective to deliver an adequate amount of riboflavin in the stroma through the intact epithelium, as previously discussed [21, 22].

The present RCT with identifier code NCT02117999 was designed to compare the treatment efficacy for progressive keratoconus by transepithelial corneal cross-linking using iontophoresis with the standard corneal cross-linking procedure at 12 months. In this paper, we present the technique and anticipate the outcomes from the complete cohort of 34 eyes after 6 months of follow-up.

## 2. Patients and Methods

### 2.1. Study Design

This is an ongoing prospective, unmasked, randomized controlled trial (RCT) conducted at the clinical trials center of the IRCCS Fondazione G.B. Bietti, Rome, Italy. The aim of the study is to assess the efficacy and safety of transepithelial corneal cross-linking using iontophoresis (T-ionto CL or study group) in the treatment of progressive keratoconus in comparison with standard corneal cross-linking (standard CL or control group). The primary outcome measure of the study is the maximum simulated keratometry value (*K*_max_) at 12 months. Approval was obtained from the IFO-IRCCS Ethical Committee (Rome, Italy), and the conduct of the study adhered to the tenets of the Declaration of Helsinki. The trial is registered with the US National Institutes of Health registry with identifier code NCT02117999 (https://clinicaltrials.gov/ct2/show/NCT02117999). After full explanation of the protocol, written informed consent was obtained from all participants before enrolment in the study. The recruitment started on January 31, 2014, and closed on May 30, 2015.

### 2.2. Participants

Patients with a confirmed diagnosis of progressive keratoconus were invited to participate in this study. Keratoconus was deemed to be progressive if there was an increase of at least 1 diopter (D) in *K*_max_ derived by computerized Placido disk corneal topography over the preceding 12 months. Exclusion criteria included a minimum corneal thickness of less than 400 *μ*m, *K*_max_ steeper than 61 D, any corneal scarring, previous refractive or other corneal or ocular surgeries, and other ocular disorders (e.g., cataract, glaucoma, and herpetic keratitis). Patients who were pregnant or breastfeeding at the time of enrolment also were excluded. Only patients aged between 18 and 46 years were included in the study.

Eligible patients were randomized after enrolment, with allocation ratio of 2 : 1, into either the study or control group using a computer-generated randomization plan with block randomization in groups of four. Two different blocks were created, which included eyes with *K*_max_ steeper or flatter than 54 D in order to randomize patients with similar baseline *K*_max_ values in the study and control groups. If both eyes of a patient qualified for participation in the study, each eye was randomized independently. Second eyes were treated no earlier than 2 months after the first eyes.

### 2.3. Assessments

Contact lens wearers were instructed to discontinue their use for a minimum of 3 weeks before the preoperative eye examination. In addition, we asked all those patients to discontinue the use of contact lens during follow-up in order to avoid bias during the study.

At baseline and postoperative visits at 3 and 7 days and 1, 3, and 6 months, all patients underwent slit-lamp examination of the anterior segment of the eye; the haze in the anterior stroma was graded on a scale (grade 0–4) used after photorefractive keratectomy [23]; ocular surface inflammation was graded on signs of bulbar conjunctival hyperemia (grade 0–3) and upper tarsal conjunctival papillae (grade 0–3) according to Akpek et al. [24]. In addition, the following assessments were recorded: best spectacle corrected visual acuity (BSCVA, logMAR units) obtained using Early Treatment Diabetic Retinopathy Study chart at 4 meters, contrast sensitivity function (CSF, log units) evaluated using Pelli-Robson chart, manifest refraction (expressed as spherical equivalent, diopters, D), *K*_max_ (D) and corneal thickness (micrometers, *μ*m) using combined Placido disk corneal topography and anterior segment optical coherence tomography (Visante, Carl Zeiss Meditec Inc., Dublin, CA, USA), and endothelial cell density (ECD, cells/mm^2^) measured by no-contact specular microscopy (Perseus, CSO, Italy). All data were acquired and analyzed in an unmasked manner. At each time point, patients received a questionnaire to evaluate symptoms, such as itchiness, tearing, photophobia, and pain (grade 0–3), after treatment.

To exclude infection, document epithelial healing, and provide general postoperative care, all patients were assessed also on day 1 after treatment in addition to the described follow-up schedule.

To improve the reliability of topography measurements, a minimum of 3 acquisitions were performed for each eye at each time interval. If the value varied by more than 10% between the scans, then a further scan was obtained. The best scan was then selected for analysis.

### 2.4. Techniques and Treatments

**Figure 1:**
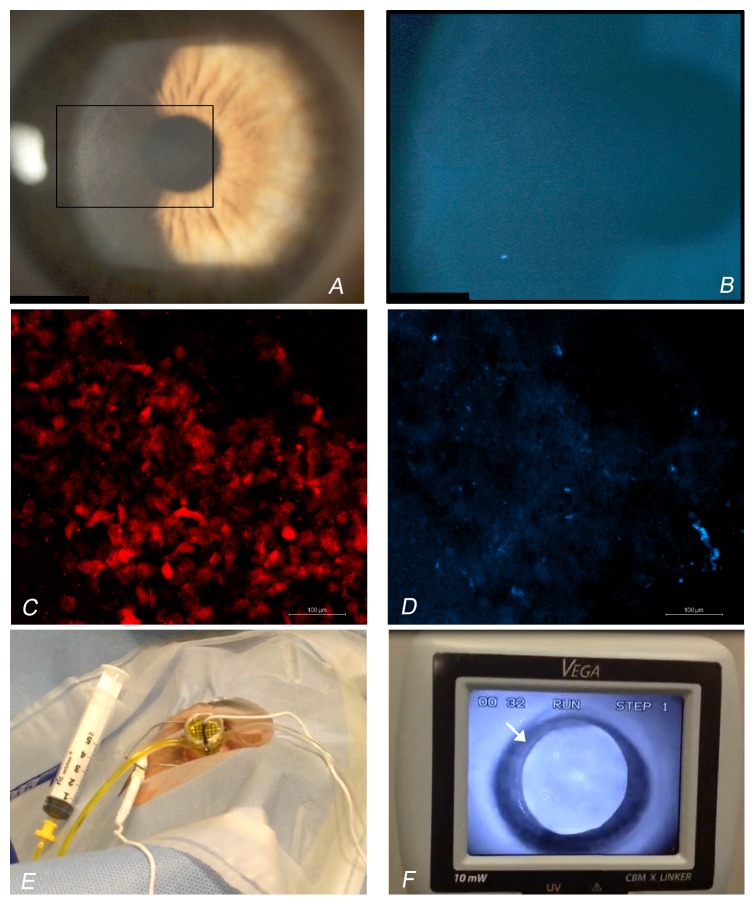
(A) Slit-lamp photograph of the cornea 1 minute after the applanation with the Biopore membrane. (B) The corneal epithelium showed moderate punctate staining with fluorescein dye (molecular weight: 376 Da). (C) Homogeneous immunofluorescent staining for MUC4 mucin (false color red) was found on Biopore membranes where the cornea was applanated. (D) A small number of scattered epithelial cells (false color blue) were observed on the membranes. In (C) and (D), scale bars are 100 *μ*m. (E) Iontophoresis was performed with the current intensity set at 1.0 mA for 5 minutes using a commercial device. (F) After iontophoresis, the cornea was irradiated using 10 mW/cm^2^ UV-A device for 9 minutes. The arrow indicates the mark of the suction tube on the corneal epithelium. Strong fluorescence was emitted by stromal riboflavin inside the area of iontophoresis delivery.

For each patient, corneal cross-linking was performed within 4 weeks of the baseline examination. All treatments were performed under topical anaesthesia; anaesthetic eye drops (oxybuprocaine hydrochloride 0.4%, Novesina, Novartis Farma SpA, Italy) were instilled 3 times over a 10-minute period before each treatment. Transepithelial corneal cross-linking using iontophoresis was performed as follows (Figure 1):

(1) After a lid speculum was inserted, central corneal thickness was measured by handheld ultrasound pachymeter (Pachmate, DGH, Exton, USA); thereafter, sterile Biopore membrane attached to a plastic cylinder (Millicell, cod. PICM01250, Merck SpA, Italy) was pressed against the central cornea with sufficient pressure to applanate the central cornea for 3 seconds and remove the precorneal mucin layer.
(2) Corneal soaking with ETDA and trometamol enriched riboflavin-5-phosphate 0.1% hypotonic solution (Ricrolin+, Sooft Italia SpA, Italy) was performed using a commercial iontophoresis device (Iontophor CXL, Sooft Italia SpA, Italy). The passive electrode was applied to the forefront of the eye to be treated. The active electrode, a bath tube made of plastic, was applied to the corneal surface. After suctioning of the tube to the corneal epithelium, it was filled with riboflavin solution. The current intensity was set at 1.0 mA for 5 minutes. After iontophoresis, the corneal surface was gently washed with chilled 0.9% sodium chloride solution.
(3) Immediately after iontophoresis, the central corneal thickness was again measured by handheld ultrasound pachymeter.
(4) Corneal UV-A irradiation was then applied using 10 mW/cm^2^ device (370 ± 8 nm; Vega 10 mW, CSO, Italy) at 56 mm distance for 9 minutes. One drop of chilled 0.9% sodium chloride solution was applied over the corneal epithelium every 3 minutes during irradiation.

The control group received conventional corneal cross-linking according to the “Dresden protocol” [3, 4]. The central 10 mm corneal epithelium was removed using Amoils' brush (Innovative Excimer Solutions Inc., Toronto, ON); central stromal thickness was then measured by handheld ultrasound pachymeter. A solution containing 20% dextran-enriched 0.1% riboflavin (Ricrolin, Sooft Italia SpA, Italy) was instilled every 3 minutes for 30 minutes before UV-A irradiation. After corneal soaking, the stromal surface of each tissue was gently washed using chilled 0.9% sodium chloride solution; thereafter, the central stromal thickness was measured by handheld ultrasound pachymeter. The corneal stroma was then irradiated with a UV-A device (Vega 3 mW, 370 ± 8 nm) with an irradiance of 3 mW/cm^2^ for 30 minutes. The UV-Adelivery system was located 56 mm from the cornea. Diluted riboflavin (0.025%) drops were instilled over the stromal surface every 3 minutes during UV-A irradiation.

The UV-A devices were calibrated with a power meter before corneal irradiation and an irradiation area of 9.00 mm diameter was used in all cases.

At the end of treatments, 2 drops of ofloxacin 0.3% (Monofloxofta, Sooft Italia SpA, Italy) were applied in all cases. A bandage contact lens was applied only to patients treated by conventional corneal cross-linking; it remained in place until epithelial closure was confirmed. After surgery, all patients continued taking ofloxacin 0.3% 5 times daily for 6 days, sodium hyaluronate 0.10% (Ribolisin, Sooft Italia SpA, Italy) 6 times daily for 3 months, and fluorometholone acetate 0.1% (Fluaton, Bausch & Lomb, Rochester, NY) 2 times daily from day 7 to day 21.

### 2.5. Immunofluorescence Microscopy Imaging

Immediately after the applanation, the Biopore membranes were fixed in 4% paraformaldehyde and shipped to the laboratory. Each membrane was gently removed from the plastic cylinder and placed into the wells of a multiwell plate. The samples were blocked in 1x phosphate buffered saline containing 1% bovine serum albumin (BSA; Sigma-Aldrich, Saint Louis, MO) and then incubated with antibody against mucin-4 (MUC4, goat polyclonal, 1 : 100; Santa Cruz Biotechnology, Santa Cruz, CA) for 2 hours and with rhodamine-conjugated secondary antibodies (anti-goat produced in donkey, 1 : 200; Sigma-Aldrich, Saint Louis, MO) for 1 hour at room temperature. In addition, the cell nuclei were stained with far-red fluorescent DNA dye (DRAQ5®, 1 : 2000; Cell Signaling Technology, Boston, MA) for 10 minutes at room temperature. Specimens were then mounted in Dako Glycergel mounting medium (Dako, Glostrup, Denmark) for immunofluorescence microscopy imaging. Images were acquired using a Nikon A1Rsi+ confocal laser scanning microscope equipped with NIS-Elements Advanced Research software (Nikon Instruments Inc., Melville, NY).

### 2.6. Statistical Analysis

Statistical analysis was performed using SPSS (version 17, IBM Corp., NY). All data are reported as the mean ± standard deviation. Normal data distribution was tested by using the one-sample Kolmogorov-Smirnov test. Sample size calculation was performed to detect a difference of 0.95 D between the mean *K*_max_ changes for the T-ionto CL and standard CL groups at 12 months, at a significance level of 5% and power of > 80%, assuming a standard deviation of 1.20 D. The sample size of the study was 34 cases (allocation ratio of 2 : 1).

In this work, the difference from baseline for each parameter was calculated at each time point (3 days, 7 days, and 1, 3, and 6 months) for each eye. The differences within each group were compared using paired Student's *t*-test. These changes were also compared between the study and control group using unpaired Student's *t*-test.

## 3. Results

Thirty-four eyes of 25 patients were randomized to T-ionto CL (20 patients, 22 eyes) and standard CL (10 patients, 12 eyes) treatments. The demographic data showed a strong skew toward male patients (20; 80%); the mean age was 31.05 ± 6.64 years and 29.40 ± 5.60 years in the study and control group (*P* = 0.55), respectively. Eleven right eyes (50%) and eleven left eyes (50%) were treated by T-ionto CL; seven right eyes (60%) and five left eyes (40%) were treated by standard CL (Table 1).

Nine patients were treated in both eyes: two patients underwent T-ionto CL in both eyes (A100 and A500; A1600 and A2000); two patients underwent standard CL in both eyes (B200 and B500; B300 and B400); and five patients received T-ionto CL in one eye and standard CL in the fellow eye (A900 and B1100; A1300 and B1200; A1400 and B600; A1500 and B700; A1800 and B900). All patients completed the 6-month follow-up.

### 3.1. Technique

Immediately after corneal applanation with a Biopore membrane, the corneal epithelium was moderately stained with fluorescein dye (Figures 1(A) and 1(B)). The Biopore membranes, which were applanated to the cornea, showed confluent staining for MUC4 mucin and the presence of scattered epithelial cells in all cases (Figures 1(C) and 1(D)).

In the study group, two eyes (10%) had perilimbal haemorrhage after removing the iontophoresis tube. This was likely related to peripheral corneal suctioning of the tube. One eye had a small central epithelial defect (<2 mm) at the end of the procedure. This was the first case in the series (A100). A bandage contact lens was applied for 24 hours and then removed once the epithelium was intact. In controls, epithelial closure was completed and the bandage contact lens was removed at day 3 in all eyes.

In the study group, central corneal thickness ranged between 491 ± 35 *μ*m and 492 ± 40 *μ*m (0.2% average change) before and after iontophoresis, respectively. In the control group, central stromal thickness decreased from 464 ± 34 *μ*m to 332 ± 27 *μ*m (28% average change) from before to after soaking with 20% dextran-enriched riboflavin solution. After UV-A irradiation of the cornea, the stromal thickness returned back toward baseline values (445 ± 16 *μ*m).

### 3.2. Symptoms

At day 1, controls complained of more tearing than those with eyes treated by T-ionto CL (*P* = 0.04); no significant differences for other symptoms were found between groups at day 1 postoperatively. At day 3, tearing was again greater in patients treated by standard CL than in those treated by T-ionto CL (*P* = 0.006); photophobia was also greater in patients treated by standard CL than in those treated by T-ionto CL (*P* = 0.001). No differences in pain score were found between groups at day 1 (1.9 ± 1.0 and 2.1 ± 0.9 in T-ionto CL and standard CL groups, resp., *P* = 0.71) and day 3 (0.6 ± 0.5 and 0.5 ± 0.7, resp., *P* = 0.65) postoperatively. After 1 week, there was no significant symptom complained of by any patient and no difference was found between groups.

### 3.3. Objective Evaluation

At day 1, bulbar conjunctival hyperaemia was greater after standard CL than after T-ionto CL, though without reaching statistical significance (*P* = 0.08); however, it was significantly greater 3 days (*P* < 0.001) and 1 week (*P* = 0.03) after standard CL than after T-ionto CL. Thereafter, no difference was found between groups. Upper tarsal conjunctival papillae were greater at days 1 (*P* = 0.004) and 3 (*P* < 0.001) after standard CL than after T-ionto CL. No difference was found between groups afterwards.

Two eyes (10%) and six eyes (50%) showed stromal edema in the T-ionto CL and control groups, respectively, both at 3 and at 7 days postoperatively. Thereafter, no edema was evidenced in the study group. Corneal edema, which was mostly confined to the posterior stroma, was still found in 4 eyes (33%) and 1 eye (8%) at 3 and 6 months after standard CL, respectively.

At 3 months, two eyes (10%) in the T-ionto CL showed faint corneal haze (grade: ≤0.50); at 6 months, all the eyes in the T-ionto CL group had clear cornea. In the control group, at 3 months, haze graded 0.50 in four eyes (33%) and 1.00 in two eyes (17%); at 6 months, haze graded 0.5 and 1.00 in five eyes (42%) and one eye (8%), respectively.

**Figure 2:**
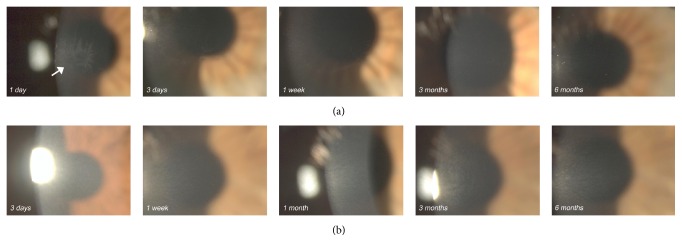
(a) Slit-lamp photographs of a representative case (A1200) after T-ionto CL. One day after treatment, moderate epithelial haze of the central cornea (arrow), with no fluorescein staining, was noted in 5 cases (23%); this superficial haze regressed in all cases within 1 week after treatment. Six months after T-ionto CL, the cornea was clear in all cases. (b) Slit-lamp photographs of a representative case (B700) after standard CL. Six months after treatment, anterior corneal haze and mild stromal edema, which was confined to the posterior stroma, were still observed in 50% and 8% of controls, respectively.

Representative pictures of study and control eyes over 6 months of follow-up are shown in Figure 2.

### 3.4. Topography Measurements

**Figure 3:**
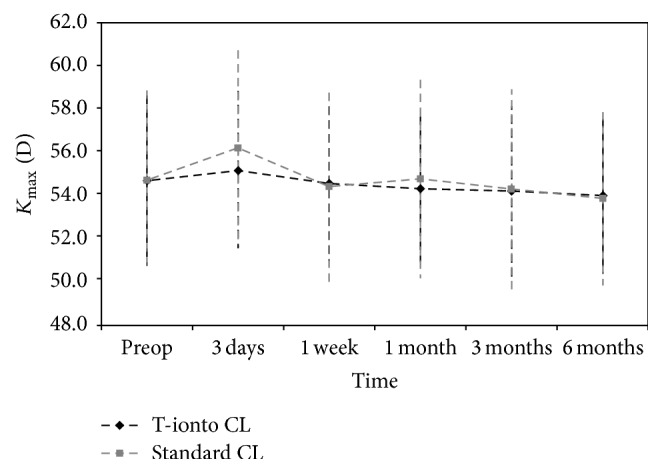
Graph showing *K*_max_ readings (D) compared with baseline at 3 days, 1 week, and 1, 3, and 6 months after T-ionto CL (black curve) and standard CL (grey curve). Bars indicate ± standard deviation. On average, both procedures halted progression of keratoconus during 6 months of follow-up.

**Figure 4:**
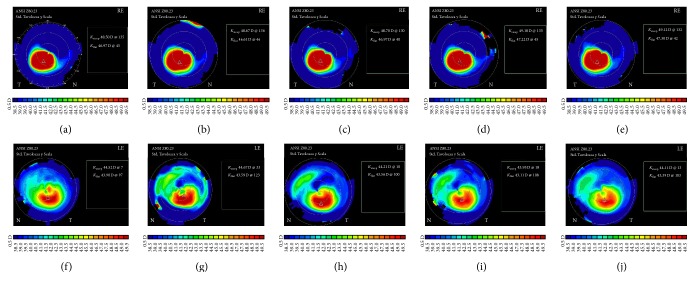
Tangential corneal topography maps in a patient who underwent transepithelial corneal cross-linking in the right eye (A1500) and standard cross-linking in the left eye (B700). The preoperative, 1-week, and 1-, 3-, and 6-month postoperative maps are shown from the left to right, respectively. At 6 months, the flattening of *K*_max_ was 1.74 D (from 59.46 D to 57.72 D) and 0.92 D (from 50.55 D to 49.63 D) in the right and left eye, respectively. Scale bars are normalized and shown in diopters. RE: right eye; LE: left eye; *K*_steep_ and *K*_flat_ represent the simulated keratometry values.

The mean increase of *K*_max_ was 2.88 ± 2.20 D and 2.78 ± 1.87 D in the T-ionto CL and standard CL groups in the preceding 12 months, respectively. At baseline, the difference in *K*_max_ between groups was not statistically significant (T-ionto CL: 54.7 ± 4.0 D; standard CL: 54.7 ± 4.3 D; *P* = 0.87). At 6 months, there was a significant average improvement in both groups with a flattening of *K*_max_ by 0.72 ± 1.20 D (*P* = 0.01) and 0.86 ± 0.89 D (*P* = 0.006) in the T-ionto CL and standard CL groups, respectively (Figure 3). Flattening of more than 1.00 D occurred in 7 eyes (32%) and 5 eyes (42%) in the T-ionto CL and control group, respectively. Two eyes (9%; A1000 = 1.2 D and A2200 = 1.2 D) of the T-ionto CL group showed progression of *K*_max_ more than 1.00 D during the same period; no eye showed progression in the standard CL group. Comparing the *K*_max_ changes between groups revealed no statistically significant differences (*P* = 0.72) at 6 months (Figure 4).

### 3.5. Visual Acuity and Refractive Outcomes

**Figure 5:**
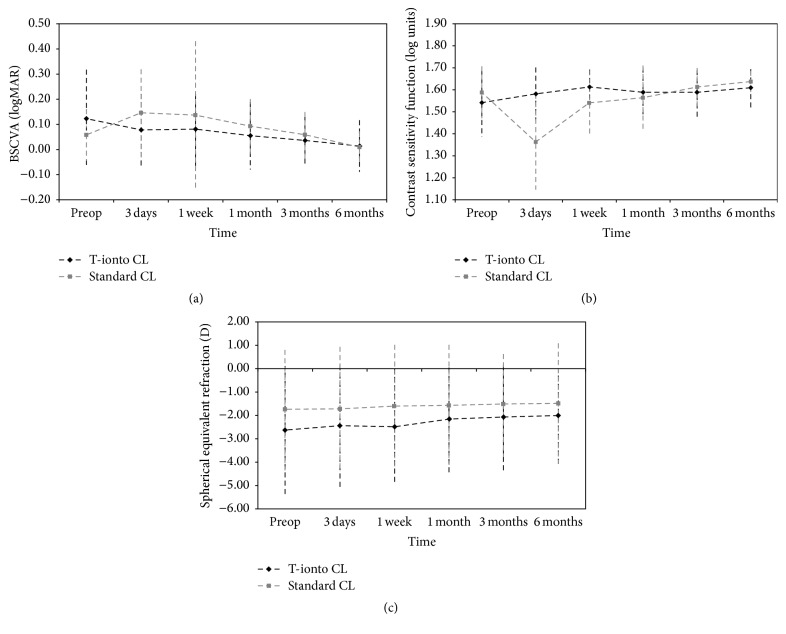
((a), (b), and (c)) Graphs showing best spectacle corrected visual acuity (BSCVA, LogMAR), contrast sensitivity function (CSF, log units), and spherical equivalent refraction (D) compared with baseline at 3 days, 1 week, and 1, 3, and 6 months after T-ionto CL (black curve) and standard CL (grey curve), respectively. Bars indicate ± standard deviation. BSCVA improved significantly (*P* = 0.08) and spherical equivalent refraction was significantly less myopic (*P* = 0.02) after T-ionto CL. On average, visual performance decreased in the first week after standard CL and reached preoperative values at 3 months postoperatively.

At baseline, the difference in BSCVA between groups was not statistically significant (*P* = 0.29). On average, BSCVA improved compared with baseline at 6 months in the T-ionto CL group (from 0.12 ± 0.20 logMAR to 0.01 ± 0.10 logMAR; *P* = 0.001). In the control group, the 6-month improvement of BSCVA compared with baseline approached statistical significance (from 0.06 ± 0.10 logMAR to 0.01 ± 0.07 logMAR; *P* = 0.08; Figure 5(a)). At 6 months, BSCVA improved by 1 or more ETDRS line in fourteen eyes (64%) and four eyes (33%) in the T-ionto CL and control group, respectively. The changes of BSCVA between the two groups did not show statistical significance (*P* = 0.19) at 6 months.

The contrast sensitivity function recovery was slower in the control than in study group (*P* < 0.001 at day 3). In the study group, CSF did not change during follow-up (preoperative range: 1.20–1.65 log units; 6-month range: 1.35–1.65 log units); in the control group, the postoperative values decreased immediately after treatment (from 1.59 ± 0.12 to 1.36 ± 0.22 log units at day 3, *P* < 0.001), approaching baseline values at 1 week (1.54 ± 0.14 log units; Figure 5(b)). After 6 months, CSF improved by one triplet in seven eyes (32%) and three eyes (25%) in the T-ionto CL and control group, respectively.

The manifest spherical equivalent refraction changed averagely by +0.65 ± 1.20 D (*P* = 0.02) and +0.24 ± 0.77 D (*P* = 0.32) in the T-ionto CL and standard CL groups, respectively, at 6 months. No significant difference in the change of manifest refraction was found between groups (*P* = 0.30) during 6 months of follow-up (Figure 5(c)).

### 3.6. Corneal Thickness Measurements

**Figure 6:**
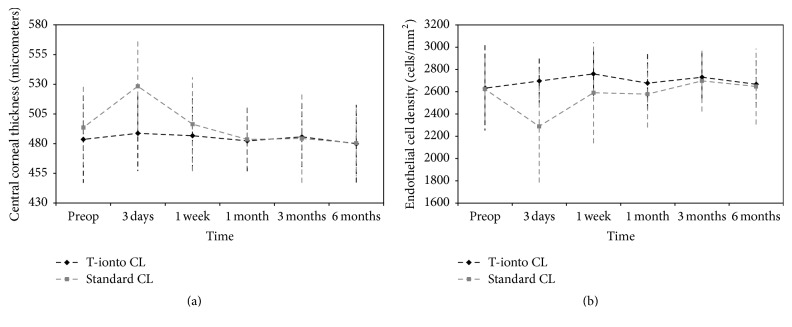
((a) and (b)) Graphs showing central corneal thickness (CCT, *μ*m) and endothelial cell density (ECD, cells/mm^2^) compared with baseline at 3 days, 1 week, and 1, 3, and 6 months after T-ionto CL (black curve) and standard CL (grey curve), respectively. Bars indicate ± standard deviation. No significant changes of CCT and ECD were found after T-ionto CL during 6 months of follow-up. Major differences between treatments in the early postoperative period are due to consequences of epithelial debridement and direct UV-A irradiation of the stroma thinned by hypertonic riboflavin solution.

At baseline, the mean central corneal thickness (CCT) was 484 ± 37 *μ*m and 494 ± 34 *μ*m in the T-ionto CL and standard CL group (*P* = 0.44), respectively. At 6 months, no significant CCT differences were found in the T-ionto CL (480 ± 33 *μ*m; *P* = 0.50), while significant corneal thinning was found in the control group (481 ± 29 *μ*m; *P* = 0.03) with respect to baseline (Figure 6(a)). On average, corneal thickness significantly increased at 3 days (*P* = 0.001) after standard CL, approaching baseline values at 1 week and slightly progressing to decrease over 6 months.

### 3.7. Endothelial Cell Density

The ECD ranged from 2635 ± 387 cells/mm^2^ and 2625 ± 281 cells/mm^2^ preoperatively to 2666 ± 235 cells/mm^2^ and 2647 ± 351 cells/mm^2^ 6 months postoperatively in the T-ionto CL (*P* = 0.66) and standard CL (*P* = 0.68) group, respectively. In the control group, ECD dropped at 3 days postoperatively (*P* = 0.03; Figure 6(b)), likely related to the loss of corneal transparency due to stromal edema found in 50% of eyes at the same visit.

### 3.8. Adverse Events

One eye in the standard CL group (B600; left eye) showed two small peripheral subepithelial infiltrates at day 3, which did not delay epithelial wounding and did not affect visual acuity. The anterior chamber was clear. The eye was treated with application of netilmicin sulfate 0.3% (Nettacin, Sifi SpA, Italy) and ciprofloxacin chlorhydrate 0.3% (Oftacilox, Alcon SA, Puurs, Belgium) drops 5 times daily each for 7 days. Fluorometholone acetate 0.1% was initiated 1 week later than scheduled in the study protocol. By 3 months, there were only two faint corneal scars, with visual performance being stable (BSCVA = 20/20 and CSF = 1.65 log units). No postoperative complications occurred in the right eye of the same subject (A1400) that underwent T-ionto CL.

## 4. Discussion

In this work, we reported the surgical techniques and preliminary clinical findings of the RTC with code identifier NCT02117999 comparing transepithelial corneal cross-linking with iontophoresis and conventional corneal cross-linking on all enrolled patients having completed 6 months of follow-up.

The eyes recruited in both arms of the present study showed similar progression of *K*_max_ in the preceding 12 months, with mean steepening of 2.88 ± 2.20 D and 2.78 ± 1.87 D in the T-ionto CL and standard CL groups, respectively. Because the main treatment objective is to stabilize the underlying disease process, corneal topography (*K*_max_) at 12 months was considered the key outcome measure of the study [1, 2]. Due to the inherent clinical interest in novel surgical techniques for treating progressive keratoconus, we are anticipating the 6-month clinical outcome.

At 6 months, we found statistically significant flattening of *K*_max_ by 0.72 ± 1.20 D (*P* = 0.01) and 0.86 ± 0.89 D (*P* = 0.006) in the T-ionto CL and standard CL groups, respectively, with no difference between groups, though two eyes in the study group (9%) showed progression of *K*_max_ of 1.2 D during the same period. Less variability and more favorable outcomes have been in general reported for standard cross-linking from the 6-month follow-up onwards, due to epithelial-stromal remodelling [2, 3, 8, 12, 25, 26]; for this reason, the design of the present RCT did not include any further treatment before completing the 1-year follow-up. Retreatment of an ectatic cornea has been previously indicated if the *K*_max_ value increased by at least 1.0 D over 2 consecutive follow-up visits compared with its value during the steady-state period after the first treatment [3].

Visual performance was not affected by T-ionto CL in the first days after surgery; at 3 days postoperatively, all eyes except for two (90%) had the same or improved BSCVA and CSF with respect to baseline. The same result was found in 50% of cases treated by standard CL in the same period. The differences between treatments were mostly related to epithelial debridement and wound healing. Six months after T-ionto CL, we found a significant average improvement in BSCVA (−0.11 logMAR; *P* = 0.001), which was not found after standard CL. No change in CSF was found after T-ionto CL (mean changes lower than +0.08 log units during a period of 6 months), whereas a decrease in CSF was measured 3 days after standard CL (on average, −0.23 log units), which was likely caused by epithelial debridement and wound healing. The manifest refraction showed a statistically significant average reduction of myopia (+0.65 ± 1.20 D; *P* = 0.02) only after T-ionto CL treatment at 6 months.

No changes in CCT and ECD were measured during 6 months of follow-up after T-ionto CL treatment. After standard CL, corneal thickness increased, due to stromal edema, during the first week postoperatively (on average, +34 *μ*m), decreasing during 6-month follow-up. A drop in ECD count, which was related to decreased transparency and more corneal scattering caused by stromal swelling in 50% of eyes, was measured at day 3 after standard CL (on average, −337 cells/mm^2^). The use of hypotonic riboflavin solution to promote stromal swelling in order to achieve a minimum thickness of 400 *μ*m before UV-A irradiation has been shown to minimize the risk of early postoperative stromal swelling [27].

Although there was no difference in pain score between groups at any time during follow-up, symptoms of ocular discomfort were greater after standard CL than after T-ionto CL during the first 3 days postoperatively. The eyes treated by standard CL showed greater conjunctival hyperaemia and tarsal conjunctival papillae than eyes treated by T-ionto CL during the first week [24]. At 3 months, 2 eyes (10%) in the T-ionto CL group showed faint corneal haze (grade ≤ 0.5), which disappeared in all cases at 6 months. In the control group, six eyes (50%) showed mild corneal haze (grade between 0.5 and 1.0) during the same period [23]. We had one case in the control group that showed peripheral sterile corneal infiltrates at day 3, which did not delay corneal wound healing or affect visual performance or topography outcome (*K*_max_ flattened by −0.8 D at 6 months). No adverse events were recorded in the study group.

Data from the control group are consistent with those already published in the literature showing the time course of clinical and instrumental measures after standard CL [2–5, 9, 25]. In general, visual acuity and corneal steepness worsen in the first month postoperatively; resolution to baseline occurs by approximately 3 months, with improvement thereafter. Improvement includes flattening of *K*_max_ (on average between 1 and 2 D in averagely 50% of cases), reduction in myopic spherical equivalent (averagely between +0.2 and +0.7 D), and increase of BSCVA (≥1 Snellen line in 50% of cases), which occur during a period of 1 year after treatment [2, 3, 8, 10, 25, 26]. Central corneal thickness remains slightly decreased from baseline to 12 months after standard CL and then recovers to baseline thickness after more than 18 months [8]. Early postoperative complications after standard CL include stromal swelling (averagely 70% of cases) and sterile infiltrates (averagely 8% of cases), which however have been shown to resolve in the vast majority of cases within 3 months after treatment [2, 6, 28]. Stromal swelling may be due to epithelial debridement, excessive stromal thinning due to the hyperosmolar riboflavin solution, and the direct UV-A irradiation of the corneal stroma. The mechanism underlying sterile infiltration is still unknown but may relate to an altered immune response to antigens in areas of static tear pooling beneath the bandage contact lens or may be a direct result of the phototoxic effect of corneal cross-linking [28, 29]. Corneal haze is also a common adverse event in the early postoperative period after standard CL (up to 70% of cases), which however resolves in the vast majority of cases by 1 year after treatment [28, 30, 31].

In this RCT, the major technical differences between the T-ionto CL and standard CL treatments include the epithelial debridement in the latter case, the use of two different riboflavin solutions, and the total UV-A energy dose delivered to corneal stroma, which is on average 20% higher in standard CL than in T-ionto CL, due to the UV-A filtering effect of the epithelium [10].

Three previous uncontrolled studies [32–34] have reported the clinical outcome of transepithelial corneal cross-linking with iontophoresis for the management of keratoconus in adults. In general, the authors have reported an improvement in BSCVA, stable or decreased *K*_max_, and no changes in CCT and ECD count during 1-year follow-up. In two studies [33, 34], iontophoresis has been performed with the same iontophoresis device used in the present RCT. Recently, the 1-year clinical outcome of transepithelial corneal cross-linking with iontophoresis (20 eyes) to treat progressive keratoconus has been compared to the standard corneal cross-linking protocol (20 eyes). The authors have shown a significant reduction of *K*_max_ by −0.31 ± 1.87 D and −1.05 ± 1.51 D, respectively, with no patient eye showing continuous progression of keratoconus at the end of follow-up [35].

In this trial, iontophoresis was preceded by removal of precorneal mucin layer with the intent to increase epithelial permeability to riboflavin (molecular weight of 340 Da) [36–39]. At physiologic pH, the intact corneal epithelial surface acts as a chemophysical barrier due to the function of the intercellular tight junctions and the epithelium-associated mucins, which regulate paracellular transport of compounds [22, 40]. By removing the epithelium-associated mucins, we aimed to decrease the epithelial barrier function in order to ensure enough bioavailability of riboflavin in the stroma using iontophoresis, as found in laboratory studies [21, 22]. Gentle applanation of the central corneal surface with Biopore membrane for 3 seconds was effective to remove the central precorneal tear film, as shown by confocal immunofluorescence analysis of the membranes containing MUC4 mucin, which is the predominant mucus moiety in the precorneal tear film [38, 39], and by corneal imaging using slit-lamp biomicroscopy and fluorescein dye (molecular weight of 376 Da). No damage to the corneal epithelium was made in any case and this clinical observation was supported by the presence of only scattered superficial epithelial cells on the Biopore membranes.

In conclusion, the preliminary outcome from the present RCT provides evidence that transepithelial corneal cross-linking with iontophoresis using 10 mW/cm^2^ UV-A device is safe for the treatment of progressive keratoconus in adults and improves keratometry readings in 90% of cases over 6 months postoperatively. Investigation of the long-term RCT outcomes is warranted to verify the efficacy of transepithelial corneal cross-linking and determine whether it may be comparable with standard corneal cross-linking in the management of progressive keratoconus.

## Figures and Tables

**Table 1:** Baseline demographics and clinical characteristics of eyes in the study (T-ionto CL) and control (standard CL) groups (M ± SD).

	T-ionto CL	Standard CL	*P* value
Age (years)	31.0 ± 6.6	29.4 ± 5.6	*P* = 0.55
Male/female gender (%)	18/3 (86%)	8/4 (67%)	
*K*_max_ (D)	54.74 ± 4.01	54.76 ± 4.30	*P* = 0.87
BSCVA (logMAR)	0.12 ± 0.20	0.06 ± 0.10	*P* = 0.29
Central corneal thickness point on AS-OCT (*µ*m)	484 ± 37	494 ± 34	*P* = 0.44
Spherical equivalent refraction (D)	−2.64 ± 2.41	−1.75 ± 2.12	*P* = 0.29
Endothelial cell density (cells/mm^2^)	2635 ± 387	2625 ± 281	*P* = 0.93

*K*_max_: maximum simulated keratometry (D: diopters); BSCVA: best spectacle corrected visual acuity (logMAR).
